# Optical multi-task learning using multi-wavelength diffractive deep neural networks

**DOI:** 10.1515/nanoph-2022-0615

**Published:** 2023-01-16

**Authors:** Zhengyang Duan, Hang Chen, Xing Lin

**Affiliations:** Department of Electronic Engineering, Tsinghua University, Beijing 100084, China; Beijing National Research Center for Information Science and Technology, Tsinghua University, Beijing 100084, China

**Keywords:** diffractive deep neural networks, multi-wavelength photonic neural networks, optical multi-task learning

## Abstract

Photonic neural networks are brain-inspired information processing technology using photons instead of electrons to perform artificial intelligence (AI) tasks. However, existing architectures are designed for a single task but fail to multiplex different tasks in parallel within a single monolithic system due to the task competition that deteriorates the model performance. This paper proposes a novel optical multitask learning system by designing multiwavelength diffractive deep neural networks (D^2^NNs) with the joint optimization method. By encoding multitask inputs into multiwavelength channels, the system can increase the computing throughput and significantly alleviate the competition to perform multiple tasks in parallel with high accuracy. We design the two-task and four-task D^2^NNs with two and four spectral channels, respectively, for classifying different inputs from MNIST, FMNIST, KMNIST, and EMNIST databases. The numerical evaluations demonstrate that, under the same network size, multiwavelength D^2^NNs achieve significantly higher classification accuracies for multitask learning than single-wavelength D^2^NNs. Furthermore, by increasing the network size, the multiwavelength D^2^NNs for simultaneously performing multiple tasks achieve comparable classification accuracies with respect to the individual training of multiple single-wavelength D^2^NNs to perform tasks separately. Our work paves the way for developing the wavelength-division multiplexing technology to achieve high-throughput neuromorphic photonic computing and more general AI systems to perform multiple tasks in parallel.

## 1 Introduction

Photonic computing utilizes photons instead of electrons for computation, which possesses the inherent advantages of light-speed processing, low-power consumption, and high-throughput capability [1–5]. Photonic neural networks (PNNs) [6–8], which implement the artificial neural network model based on photonic computing, can achieve leapfrog improvement in computing speed and energy efficiency. Therefore, it’s considered one of the most promising solutions to support the sustainable development of artificial intelligence (AI) in the post-Moore era [9, 10]. Among different PNN architectures, diffractive deep neural networks (D^2^NN) [11, 12] can achieve large-scale neural information processing and have attracted a vast amount of interest. D^2^NN consists of layers of diffractive neurons and their optical interconnections based on the diffraction of light, which can be trained with deep learning optimization methods to fit desired mapping functions between input optical fields and output detector measurements.

In the previous studies of D^2^NN architecture [], monochromatic plane waves are used to encode the input data and propagate through modulation layers, performing a specific task. Inspired by biological intelligence [17], the multiplexing of different tasks in a single D^2^NN system is of great importance in improving its generalization and expanding its applications for different scenarios. However, performing multiple AI tasks in parallel with a monolithic D^2^NN system remains challenging. One of the major obstacles is the competition among tasks during the training, which leads to catastrophic forgetting [18, 19]. Catastrophic forgetting occurs when systems are trained on multitasks, which leads to a tendency for knowledge of previously learned tasks to be abruptly lost while learning new tasks, resulting in the deterioration of performance on every single task. Previous solutions [20, 21] require the mechanical movement of optical elements to switch between tasks one at a time or require the design of multiple different D^2^NN systems, one for each task, significantly increasing the hardware complexity.

Here, we propose an optical multitask learning monolithic system design that can simultaneously perform multiple classification tasks on different databases without mechanical movement by developing multi-wavelength D^2^NNs. Different from previous broadband D^2^NNs [22, 23], the wavelength dimension is exploited in this work to improve the computing throughput, which encodes different inputs into different wavelength channels and performs photonic computing in both spatial and spectral dimensions. We demonstrate that the multi-wavelength D^2^NNs allow for high-parallel processing of multiple inputs and significantly alleviate the competition among different tasks to preserve the high performance of each task. In this design, the multi-wavelength D^2^NN has *N* (*N* ≥ 2) different parallel wavelength channels, encoding *N* different inputs in parallel, and the detection area of each category is segmented into *N* parts, where each part represents the category of input encoded at the corresponding wavelength channel. We use the multiwavelength joint optimization method with the loss functions of softmax cross-entropy and energy efficiency constraint to train the D^2^NN. We first verify the high-parallel characteristic of a three-wavelength D^2^NN by classifying three different inputs in parallel based on the MNIST database, where the accuracy at each wavelength is comparable to training three single-wavelength D^2^NN with sequential inputs. To perform multiple tasks in parallel, we encode the inputs from different databases into different wavelength channels. We utilize the two-wavelength and four-wavelength D^2^NNs for performing the two-task and four-task classifications, respectively, based on the databases of MNIST, FMNIST, KMNIST, and EMNIST. With the increase in task numbers, the multi-wavelength D^2^NNs achieve significantly higher classification accuracy than the single-wavelength D^2^NNs and maintain the model accuracy for each task with larger network sizes, demonstrating the great advantages of multiwavelength D^2^NNs in realizing optical multitask learning.

## 2 Methods

**Figure 1:**
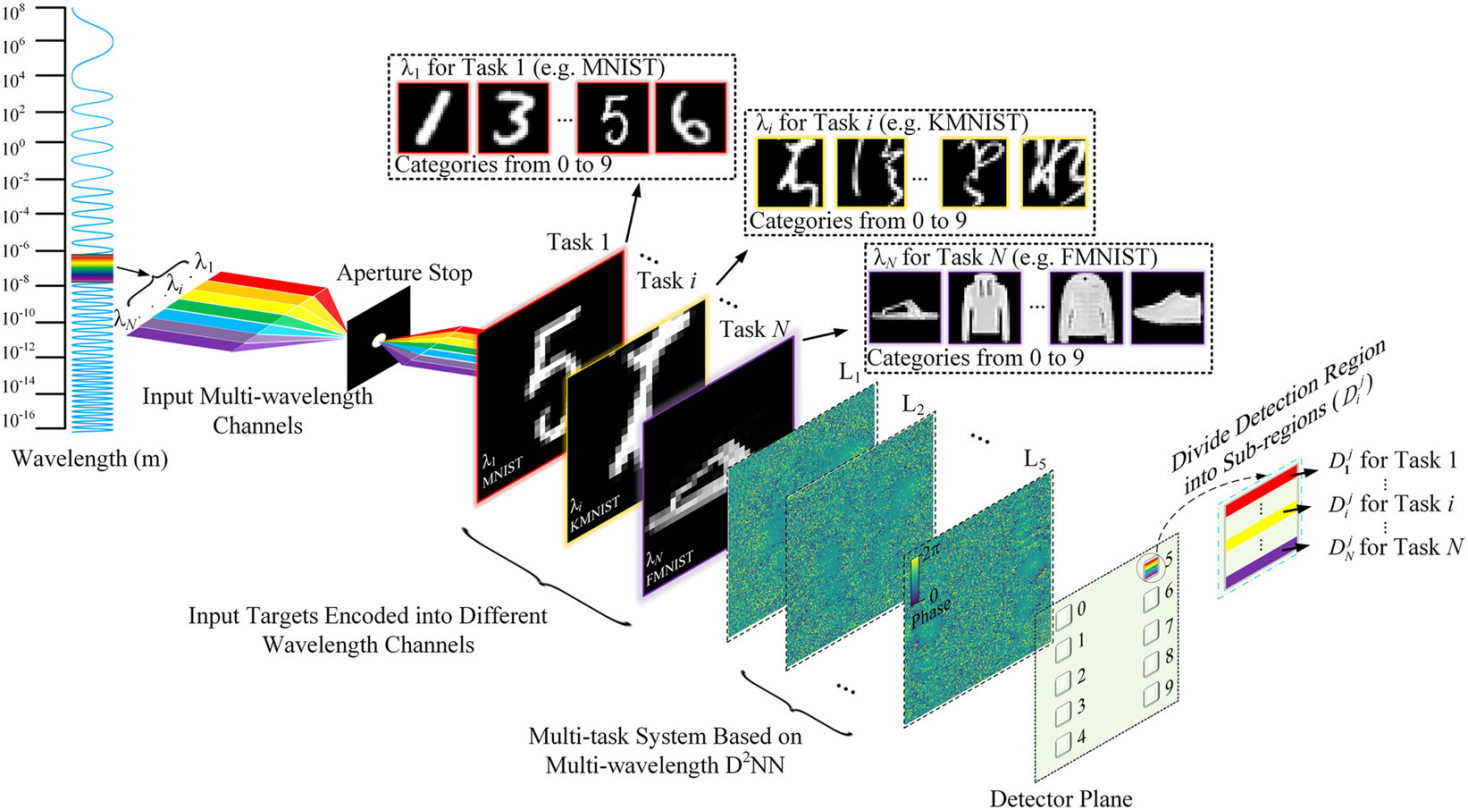
The architecture of multiwavelength D^2^NNs for optical multitask learning. The incident light comprises multiple wavelength channels, where each detection region is correspondingly segmented into multiple sub-regions. Each sub-region represents an input category type for each task.

As shown in Figure 1, the proposed optical multitask learning system achieves multiple tasks in parallel by designing a multiwavelength D^2^NN. Each wavelength channel (*λ*_*i*_, *i* = 1, … , *N*) encodes the input targets of each task *i*. By using the approximation theory of multiwavelength optical systems [24–27], the transformation of multiwavelength optical fields can be recognized as a combination of independent transformation of coherent optical fields at each wavelength, following the principle of superposition of optical intensities. The input optical fields $U_{\lambda_{i}}$ at the wavelength of *λ*_*i*_, encoding the input targets of task *i*, are transformed by the D^2^NN before the detection. We consider the linear D^2^NN at the wavelength of *λ*_*i*_ with the complex transform function of $M_{\lambda_{i}}\left(\Phi \right)$ , where **Φ** represents the learnable phase modulation coefficients of the diffractive elements at multiple phase-only diffractive layers. The detailed derivation of complex transform function can be found in [11, 12, 28]. We first assume that the phase modulation coefficients of each diffractive layer are the same under different wavelengths with the multiwavelength diffractive optical element (DOE) design (see Discussion section). Then, the output optical fields at the *i*th wavelength *λ*_*i*_ can be formulated as: $U_{\lambda_{i}}^{\prime }=M_{\lambda_{i}}\left(\Phi \right)U_{\lambda_{i}}$ , and the detector measures the intensity distribution of output optical fields that can be formulated as: $I_{\lambda_{i}}={\left|U_{\lambda_{i}}^{\prime }\right|}^{2}={\left|M_{\lambda_{i}}\left(\Phi \right)U_{\lambda_{i}}\right|}^{2}$ . For the multiwavelength D^2^NN, the total intensity distribution of different wavelengths can be formulated as the superposition of detected intensity distribution at each wavelength: $I=\underset{\lambda_{i}}{\sum }I_{\lambda_{i}}=\underset{\lambda_{i}}{\sum }{\left|M_{\lambda_{i}}\left(\Phi \right)U_{\lambda_{i}}\right|}^{2}$ .

We develop the joint optimization method to train multiwavelength D^2^NNs for performing optical multitask learning. Each category detection area at the output plane is divided into multiple sub-regions { $\left.D_{i}^{j}\right\}$ , where *i* = 1, … , *N* denotes the index of the task encoded at *i*th wavelength *λ*_*i*_; and *j* = 1, … , *M* denotes the index of detection areas, representing the index of categories. We calculate the average intensity of the *i*th sub-region among the *M* category detection areas, i.e., $P_{i}=\left\{avg\left(I\left(D_{i}^{j}\right)\right),j=1,\ldots ,M\right\}$ , where $I\left(D_{i}^{j}\right)=\underset{\lambda_{i}}{\sum }I_{\lambda_{i}}\left(D_{i}^{j}\right)$ is for the broadband wavelength detection without using spectral filters on the detector. The information encoded in different spectral channels of multiwavelength D^2^NNs is independent of each other during the diffraction propagation and modulation, where the presence of channel crosstalk only existed in the detection plane. The minimization of such channel crosstalk can be achieved by incorporating the additional wavelength selective filters or developing the multiwavelength D^2^NN training method with a joint optimization process. The wavelength selective filter can be applied to each sub-region to completely eliminate the crosstalk during intensity detections among wavelength channels for further improving the task performance. In this case, each sub-region for each task only detects the optical signals at the corresponding wavelength channel, with which $I\left(D_{i}^{j}\right)=I_{\lambda_{i}}\left(D_{i}^{j}\right)$ . To remove the filter for lower system complexity, the proposed joint optimization method during the training of multiwavelength D^2^NNs can minimize the channel crosstalk during the detection. The category type of inputs for the *i*th task is determined by finding the sub-region of the detection area with maximum average intensity, i.e., the index of the maximum value in the vector ***P***_*i*_. Besides, we further include the constraint to maximize the energy transmission efficiency of multiwavelength D^2^NNs by minimizing the optical energy outside the category detection areas. Therefore, the joint optimization problem for multiwavelength D^2^NNs training can be formulated as:

(1) $$\underset{\Phi }{\min}\left(\underset{i}{\sum }L\left(P_{i},G_{i}\right)+\underset{\lambda_{i}}{\sum }MSE\left(I_{\lambda_{i}}-\underset{j}{\sum }I_{\lambda_{i}}\left(D_{i}^{j}\right)\right)\right),$$

where $L\left(P_{i},G_{i}\right)$ represents the softmax cross-entropy loss function of *i*th task at the wavelength of *λ*_*i*_ between the detection ***P***_*i*_ and ground truth label ***G***_*i*_; ***G***_*i*_ is a one-hot vector with a length of *M*; $MSE\left(I_{\lambda_{i}}-\underset{j}{\sum }I_{\lambda_{i}}\left(D_{i}^{j}\right)\right)$ represents the total energy of optical intensity outside the sub-regions of category detection areas evaluated with the mean square error.

In the process of training, different wavelength channels share the same phase modulation coefficients at each diffractive layer that are iteratively updated to perform multitask functions by solving the joint optimization problem in Eq. (1). We use the stochastic gradient descent approach to train the multi-wavelength D^2^NNs. The input targets of the training datasets of different tasks are encoded into the amplitude of optical fields at different wavelengths to feed into the network input layer. The residual error of network outputs with respect to ground truth labels and the total optical energy outside the category detection areas are calculated according to Eq. (1), which are used to perform the error back-propagation to optimize the network structure and the phase modulation coefficients of optical diffractive elements (DOEs).

To further improve the viability of multiwavelength DOEs, another design strategy can be incorporated to optimize the relative height map Δ**Z** instead of the phase map **Φ** of DOEs during the training, with which the complex transform function of multiwavelength D^2^NNs can be changed from $M_{\lambda_{i}}\left(\Phi \right)$ to $M_{\lambda_{i}}\left(\Delta Z\right)$ . As the relative height map of each diffractive layer is the same under different wavelength channels, the phase modulation value at each wavelength is determined by the wavelength-depended material refractive index. The transformation between diffractive elements’ relative height map Δ**Z** and the wavelength-dependent phase values $\Phi_{\lambda_{i}}$ under a certain wavelength channel *λ*_*i*_ can be formulated as: $\Phi_{\lambda_{i}}=2\pi \Delta n_{\lambda_{i}}\Delta Z/\lambda_{i}$ , where $\Delta n_{\lambda_{i}}$ is the refractive index difference between the base material, e.g., the S_*i*_O_2_ and air [11, 28]. With the same training process, the numerical analysis (see Table 1) validates that both design strategies, i.e., optimizing the phase maps or height maps, for training multiwavelength D^2^NNs achieve comparable performance, where optimizing the height maps has a slightly lower classification accuracy.

**Table 1:** Performance comparisons between the multiwavelength and single-wavelength D^2^NNs for two-task classifications based on the design strategies of optimizing the phase maps or height maps of DOEs.

Diffractive neural	Diffractive neural	Accuracy by optimizing phase maps (Φ)		Accuracy by optimizing height maps (ΔZ)	
network model	network size	MNIST (Task I)	FMNIST (Task II)	MNIST (Task I)	FMNIST (Task II)
Single-task, single-wave	200 × 200 × 5	97.1%	87.5%	97.1%	87.5%
Multitask, single-wave	200 × 200 × 5	92.4%	83.1%	92.4%	83.1%
Multitask, multiwave (w/o filter)	200 × 200 × 5	95.6%	86.8%	95.2%	86.4%
Multitask, multiwave (w/Filter)	200 × 200 × 5	95.9%	87.0%	95.7%	87.0%
Multitask, multiwave (w/o filter)	400 × 400 × 5	97.5%	88.0%	97.1%	87.7%
Multitask, multiwave (w/Filter)	400 × 400 × 5	97.6%	88.9%	97.4%	88.5%

## 3 Results

### 3.1 Multiwavelength D^2^NNs for high-parallel classification

**Figure 2:**
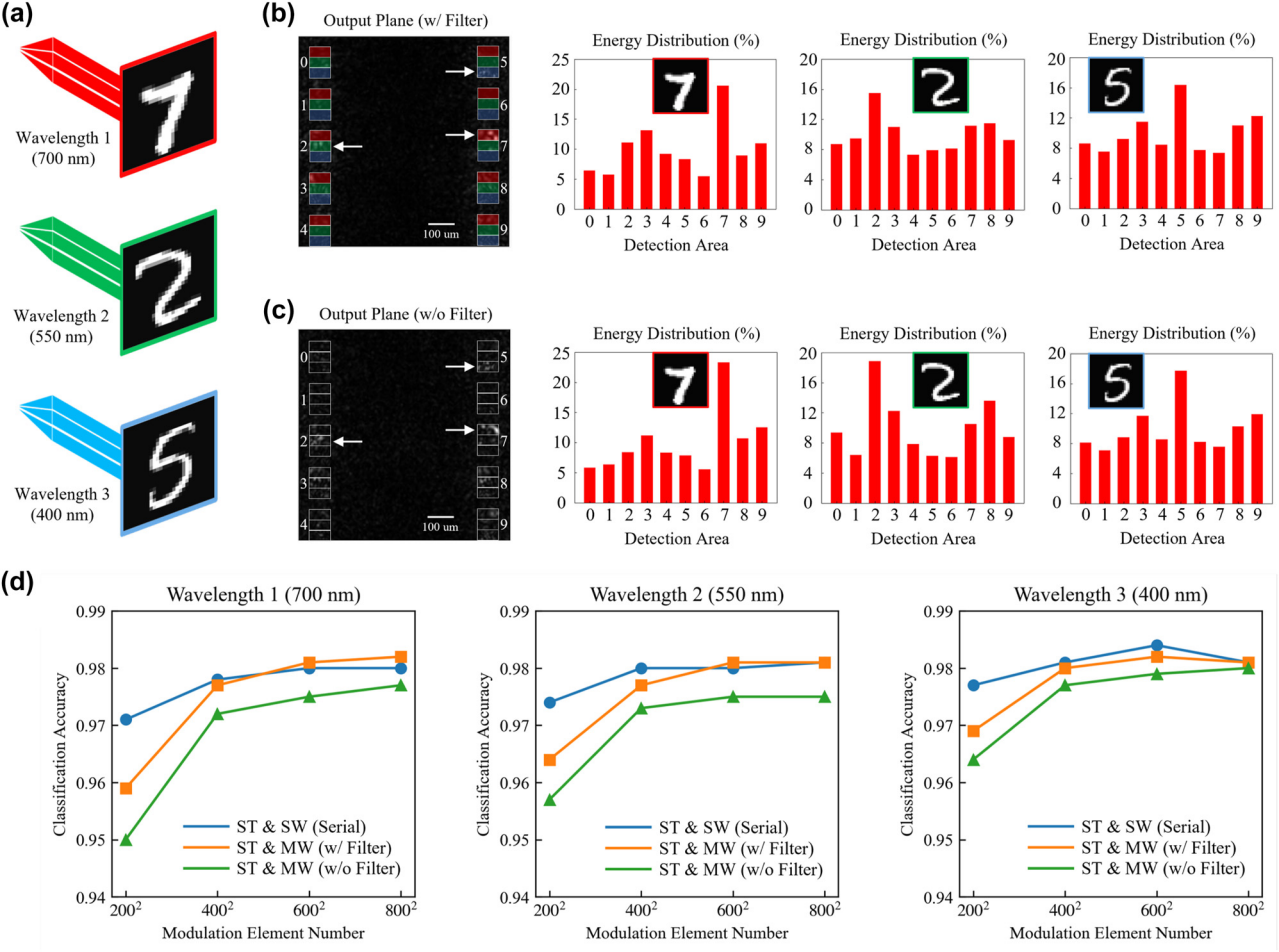
Multiwavelength D^2^NNs for high-parallel classification. (a–c) The exemplar results of simultaneously classifying three handwritten digits encoded in three wavelength channels. The classification results are evaluated with and without the wavelength selective filters on each category detection area. (d) The comparison of performances between the three-wavelength D^2^NNs for high-parallel classification and training three single-wavelength D^2^NNs with serial inputs under different network layer sizes. The results demonstrate the capability of multiwavelength D^2^NNs for high-parallel classification. ST and SW, single-task using single-wavelength with serial inputs; ST and MW, single-task using multiwavelength with parallel inputs.

We first verify the application of multiwavelength D^2^NNs for high-parallel classification that can simultaneously classify multiple inputs in performing a single task. In this work, the multiwavelength D^2^NN models were implemented using Python version 3.8.13 and PyTorch framework version 1.11.0. using a desktop computer (GeForce GTX 3090 Ti Graphical Processing Unit, GPU, and Intel (R) Xeon (R) Gold 6226R CPU @2.90 GHz and 512 GB of RAM, running an Ubuntu 20.04 operating system). To demonstrate, we build a three-wavelength D^2^NN architecture with five diffractive modulation layers, which is applied for classifying the MNIST database that can recognize three handwritten digits at each instant of time. We consider the visible wavelength ranging from 400 nm to 700 nm, where the input light source was set as the combination of three wavelengths of 400 nm, 550 nm, and 700 nm, encoding three handwritten digits, respectively. Therefore, each category detection area at the output plane is correspondingly segmented into three sub-regions. The detection region and sub-region layout have minor effects on the performance, where we adopt a similar detection region layout in [28] and separate the detection regions into sub-regions. The Adam optimizer is used for network training to optimize the phase modulation coefficients **Φ** or the relative height map Δ**Z** of the optical diffractive elements. Each optical diffractive element size was set to 4 μm × 4 μm. We first evaluate the performance of multi-wavelength D^2^NNs by setting the modulation element number at each network layer to 200 × 200, corresponding to the network layer size of 0.8 mm × 0.8 mm (see Figure 2b). We further evaluate and compare the network performance under different modulation element numbers at each layer, i.e., *K* × *K*, *K* = 200, 400, 600, and 800 (see Figure 2d). The layer number was set to 5, and the distance between successive layers was optimized according to the maximum half-cone diffraction angle theory [11, 28]. With a training batch size of 32, the initial learning rate is set to 0.01 and is reduced by half, i.e., multiplied by 0.5, after every epoch during the training. The network training converges after five epochs to achieve the desired mapping function for the multichannel inputs and output. The network was trained and blind-tested with 60,000 and 10,000 multiwavelength samples, respectively. The training and testing samples of each wavelength were constructed by randomly shuffling 60,000 and 10,000 handwritten digits from the MNIST training and testing sets, respectively. For the network layer with a modulation element number of *K* × *K*, each digit with a pixel number of 28 × 28 is resized to *K*/2 × *K*/2 and padded to *K* × *K*.

The numerical evaluation results are shown in Figure 2, where the performance of multiwavelength D^2^NNs is validated with and without wavelength selective filters on each category detection area. Figure 2a–c shows an exemplar result of simultaneously classifying three handwritten input digits, i.e., “7”, “2”, and “5”, encoded in the wavelengths of 700 nm, 550 nm, and 400 nm, respectively, under the phase modulation element numbers of 200 × 200 at each layer. The classification result of each wavelength channel is determined by finding the maximum average intensity value among the corresponding sub-regions of category detection areas, indicated with three white arrows for three input digits, as shown in Figure 2b–c, left. The energy distributions of the classification results of three inputs at different wavelength channels in Figure 2b–c show that the proposed system could prominently identify the sub-region with maximum average intensity for the correct categorization. Due to the use of wavelength selective filters to completely eliminate the wavelength crosstalk during the detection, the classification accuracies of multiwavelength D^2^NNs with wavelength selective filters are 95.9%, 96.4%, and 96.9% for the wavelengths of 700 nm, 550 nm, and 400 nm, respectively, which are slightly higher than the classification accuracies of broadband wavelength detection without wavelength selective filters, i.e., 95.0%, 95.7%, and 96.4%, respectively. For both network settings, the classification accuracies of multi-wavelength D^2^NNs further improve at each wavelength with the increase of the modulation element numbers at each network layer, as shown in Figure 2d. Under the modulation element number of 800 × 800 at each layer, the classification accuracies of multiwavelength D^2^NNs with wavelength selective filters, achieving 98.2%, 98.1%, and 98.1% for the wavelengths of 700 nm, 550 nm, and 400 nm, respectively, are comparable to training three single-wavelength D^2^NNs with the serial inputs, i.e., sequential input of digits. The results verify that multiwavelength D^2^NN can significantly increase the parallel computing capability. Using multiwavelength D^2^NNs for multitask learning by encoding different tasks into different channels enables different machine learning tasks to be implemented in parallel within a single system.

### 3.2 Optical multitask learning using multiwavelength D^2^NNs

**Figure 3:**
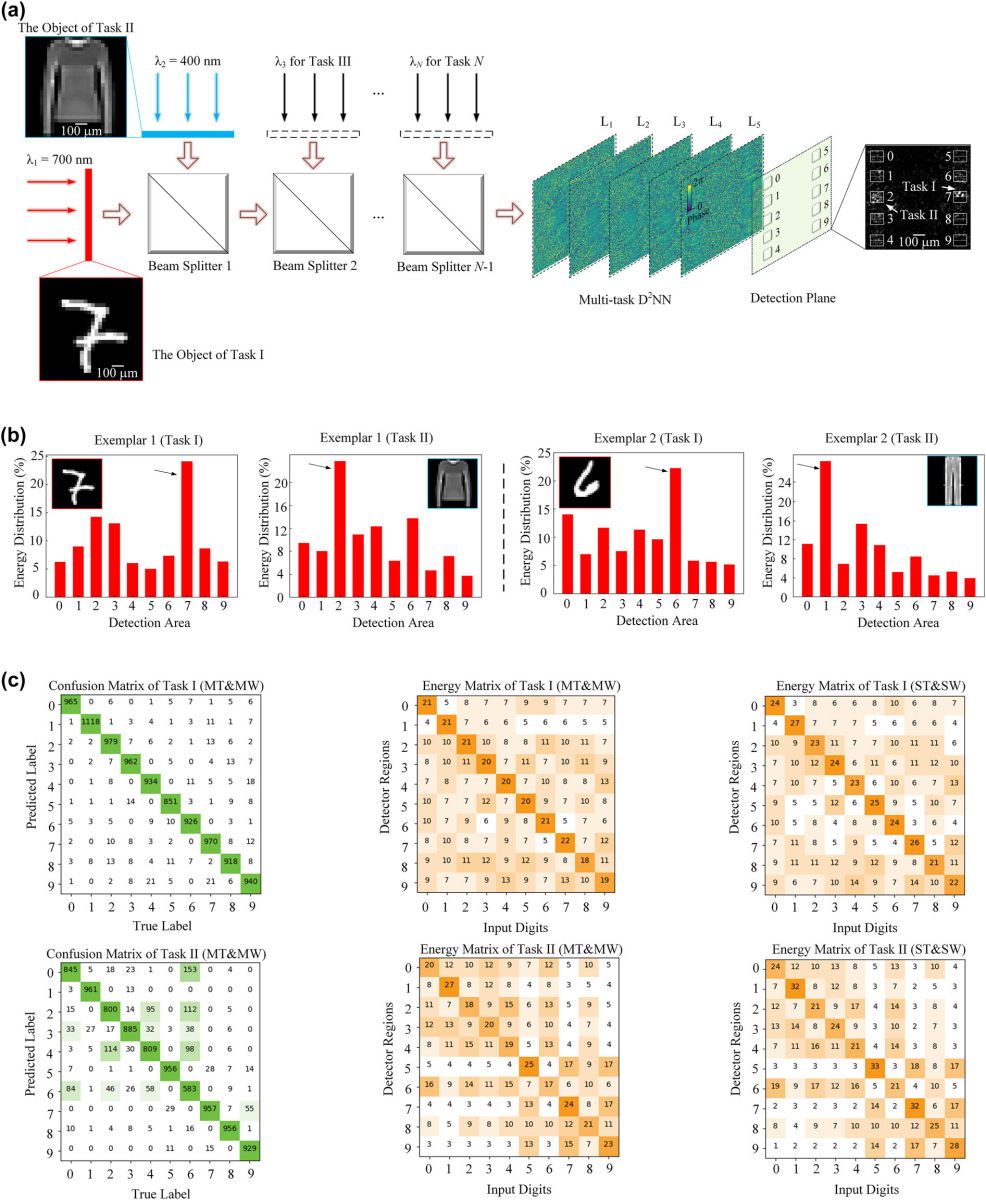
Multiwavelength D^2^NNs working under two wavelengths for classifying both the MNIST and FMNIST databases. (a) Images from the MNIST and FMNIST datasets are encoded in wavelengths of 700 nm and 400 nm, respectively. The categories of two inputs are determined by finding the corresponding sub-regions with maximum average intensity as indicated by the white arrow. (b) Energy distributions of the classification results of two tasks demonstrated the success of the proposed approach for classifying two inputs. (c, left) Confusion matrices and (c, middle) energy matrices (percentage) of two tasks, corresponding to the classification accuracies of 95.6% and 86.8% for Task I and Task II, respectively. (c, right) Energy matrices (percentage) of two single-wavelength D^2^NNs for individually performing each of the two tasks, respectively. ST and SW, single-task using single-wavelength; MT and MW, multitask using multiwavelength.

To demonstrate the capability of multiwavelength D^2^NNs for optical multitask learning, we first construct a two-task classifier for classifying both the MNIST database (task I) and the fashion-MNIST (FMNIST) database (task II). Both databases include 60,000 training samples and 10,000 testing samples with 10 category numbers. For the multiwavelength D^2^NN, the training and testing samples of each wavelength were constructed by randomly shuffling the training and testing samples from each database, respectively. Therefore, the two-wavelength D^2^NN was constructed by dividing each of 10 detector areas into two sub-regions, where the upper and lower regions represent the classification results of tasks I and II, respectively, as shown in Figure 3. The handwritten digits of task I are encoded in the wavelength of 700 nm and the fashion products of Task II are encoded in the wavelength of 400 nm. With other network settings the same in Figure 2, we first set the two-wavelength D^2^NN to have five diffractive layers, each layer with a phase modulation element number of 200 × 200, without the wavelength selective filters on the detector. Figure 3a shows an exemplar result for simultaneously classifying a handwritten digit “7” with the category number of 7 from the MNIST database and a fashion product “pullover” with the category number of “2” from the FMNIST database. The maximum average intensity outputs of task I and task II were focused on the upper sub-regions of the No. 7 detector area and the lower sub-regions of the No. 2 detector area, respectively, which were marked by the white arrow in Figure 3a. The additional exemplar results, such as a handwritten digit “6” with the category number of six and a fashion product “trouser” with the category number of “1” were also shown in Figure 3b. The energy distributions of the classification results of two tasks in Figure 3b show that the multiwavelength D^2^NN can prominently identify the sub-region with maximum average intensity for the correct categorization.

The blind testing of the trained two-wavelength D^2^NN model on the test datasets of MNIST and FMNIST achieves classification accuracies of 95.6% and 86.8%, respectively. The corresponding confusion matrices and energy distribution matrices, statistically summarizing the classification results of all samples and energy distribution percentages of two tasks, are shown in Figure 3(c, left) and Figure 3(c, middle), respectively. The average energy percentages of correct categories are 20.8% and 21.8% for two tasks, respectively. We further compare the performance of two-wavelength D^2^NNs for performing two tasks in parallel with respect to the single-wavelength D^2^NNs for performing two tasks in parallel by overlapping to multiplex two images from two datasets, respectively, as the network input, as shown in Table 1. The classification accuracies of single-wavelength D^2^NNs for performing two tasks in parallel are 92.4% and 83.1%, respectively, which is much lower than the two-wavelength D^2^NNs. We also train two single-wavelength D^2^NNs for individually performing each of the two tasks, where the classification accuracies are 97.1% and 87.5% for tasks I and II, respectively. The average energy percentages of correct categories are 24.2% and 26.1% for two tasks with two energy distribution matrices shown in Figure (3c, right), respectively. Although the two-wavelength D^2^NN have lower average energy percentages of correct categories than two single-wavelength D^2^NNs, it has the comparable energy transmission rate at the detection regions. To improve the performance of two-wavelength D^2^NN, we can increase the modulation element numbers at each layer, which achieves the classification accuracies of 97.5% and 88.0% for two tasks with 400 × 400 modulation element numbers per layer. The performance can be further improved by using the wavelength selective filters on the category detection regions, which achieves the classification accuracies of 95.9% and 87.0% for two tasks with 200 × 200 modulation element numbers per each layer and 97.6% and 88.9% for two tasks with 400 × 400 modulation element numbers per each layer, showing comparable and even higher accuracy than individually training two single-wavelength D^2^NNs to perform two tasks separately. Besides, the design strategy of multiwavelength D^2^NNs by optimizing the relative height maps instead of phase maps of DOEs achieves comparable performance with slightly lower classification accuracies in the same tasks. The results are summarized in Table 1, which verifies that the designed two-wavelength D^2^NN with a joint training approach can successfully classify the targets from two tasks in parallel without any mechanical adjusting of diffractive layers for two tasks.

**Figure 4:**
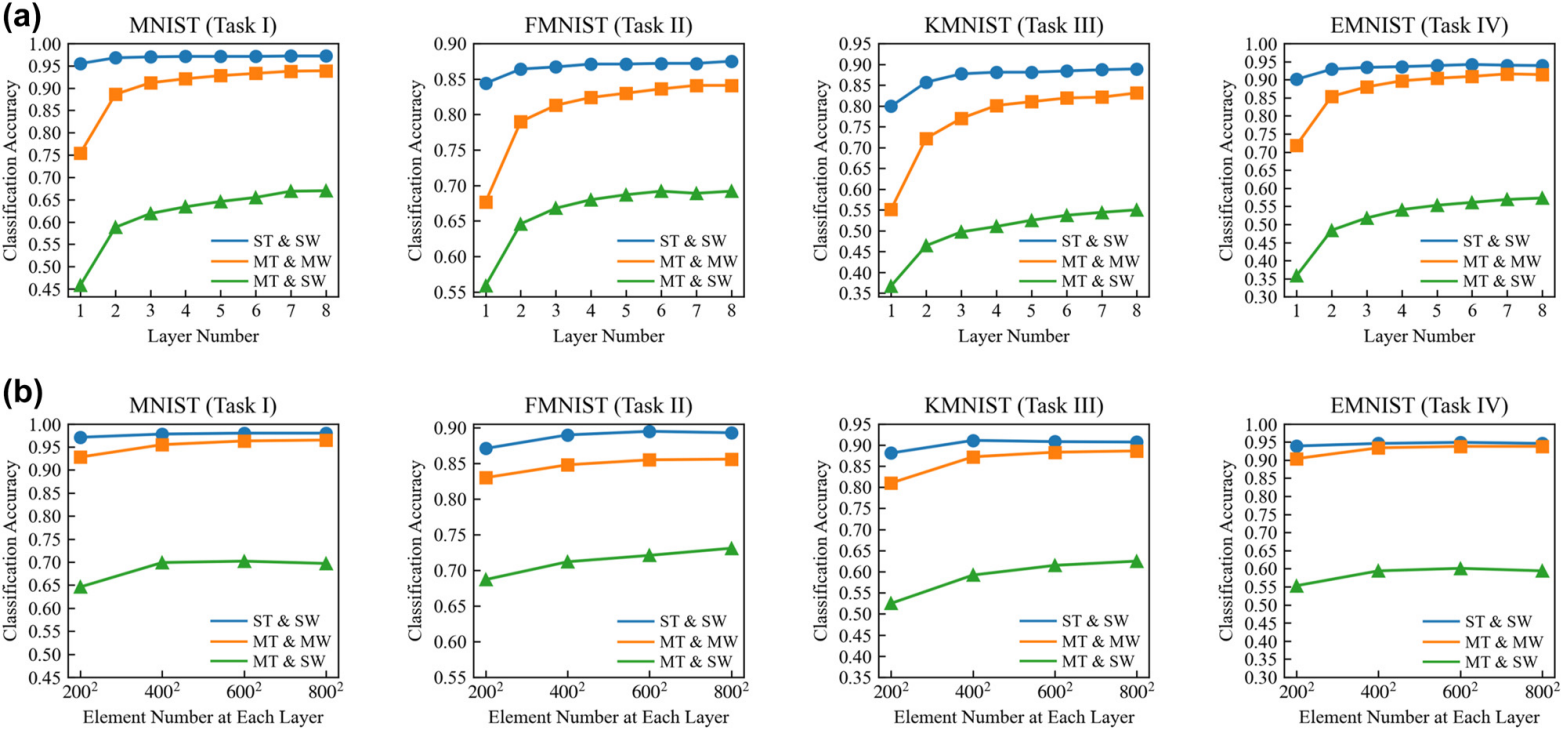
The performance of four-wavelength D^2^NNs for four-task classification without wavelength selective filters. The classification accuracies of four-wavelength D^2^NNs in performing the four-task classification are significantly higher than the single-wavelength D^2^NNs. By increasing the layer number (a) and modulation element numbers (b) at each layer, the classification accuracies of four-wavelength D^2^NNs increase at each of four tasks and approach the classification accuracies by individually training four single-wavelength D^2^NNs to perform four tasks separately. ST and SW, single-task using single-wavelength; MT and SW, multitask using single-wavelength; MT and MW, multitask using multiwavelength.

To demonstrate the capability of multiwavelength D^2^NNs for multi-task learning with more number of tasks, we constructed a four-wavelength D^2^NN for four-task classification that can simultaneously classify four targets from the databases of MNIST (task I), FMNIST (task II), Kuzushiji-MNIST (KMNIST, task III), and extended-MNIST (EMNIST, task IV), respectively. The KMNIST comprises images of ancient Japanese scripts with the same dataset size and category numbers as the MNIST and FMNIST databases. We randomly selected 10 categories of handwritten letters from the EMNIST database and kept the same dataset size as the other three tasks, i.e., 60,000 training samples and 10,000 testing samples. The databases of four tasks, from the task I to IV, are encoded in the wavelengths of 700 nm, 600 nm, 500 nm, and 400 nm, respectively. In this numerical experiment, the four-wavelength D^2^NNs are designed without using wavelength selective filters that have lower hardware complexity. With other network settings the same as Figures 2 and 3, we evaluated the classification accuracies of four-wavelength D^2^NNs in performing four tasks in parallel under different network sizes and compared the classification accuracies with the single-wavelength D^2^NNs, as shown in Figure 4. For the four-wavelength D^2^NN with five layers and the modulation element number of 200 × 200 at each layer, the classification accuracies of four tasks, from the task I to IV, are 92.8%, 83.0%, 81.0%, and 90.4% respectively, which are significantly higher than the single-wavelength D^2^NN of 64.6%, 68.7%, 52.5%, and 55.3%, under the same network size. The four-wavelength D^2^NNs for four-task classification consistently achieved much higher accuracies than the single-wavelength D^2^NNs with varying network sizes. As the number of tasks increases from two to four, the proposed multi-wavelength D^2^NN shows more advantages in realizing optical multi-task learning.

We further evaluated and compared the performance of the proposed four-wavelength D^2^NNs with respect to the individual training of four single-wavelength D^2^NNs to perform four tasks separately (see Figure 4 under different network sizes. Figure 4a increases the network size by increasing the layer numbers from one to 8 with the same element number of 200 × 200 at each modulation layer. Figure 4b increases the network size by increasing the element number at each modulation layer with the same layer number of 5. Increasing the neural network size of multiwavelength D^2^NNs for optical multitask learning can significantly improve its inference capability until the performance reaches a state of saturation. The performance of four-wavelength D^2^NNs continues to improve with the increase of network size and approaches to the performance of training four single-wavelength D^2^NNs. The classification accuracies of task I to task IV are 96.5%, 85.6%, 88.6%, and 93.8%, respectively, with the modulation layer number of five and the element number of 800 × 800 at each layer, which shows comparable performance with respect to the training of four single-wavelength D^2^NNs with the same network size. The results demonstrate the effectiveness of the proposed approach for multi-task learning with a monolithic optical system and achieve much lower hardware complexity. The encoding of multitasks into multiwavelength channels alleviates the competition among different tasks and minimizes the performance reduction of each task.

## 4 Discussion

There are different methods that have been widely validated for fabricating multiwavelength DOEs. We have demonstrated two design strategies for training multiwavelength D^2^NN by optimizing the height maps or phase maps of DOEs for each diffractive layer. With the height maps of DOEs, a multistep photolithography-etching process can be used as a viable fabrication method that has been extensively studied [28]. With the phase maps of DOEs, the same phase modulation characteristic under different wavelength channels can be achieved [29–34]. The geometry structure of each element can be determined to make the optical path length of each modulation element have the same phase value for each wavelength. This can be achieved by adding an integral multiphase delay (e.g., 2π) to one wavelength until the other wavelength reaches the appropriate phase retardation [29, 30]. The overall physical height will be based on the actual accuracy requirements. The other method exploits the refractive index change of dispersive materials for different wavelengths. One can control the optical path length of each wavelength [31]. A multiwavelength DOE can also be designed by combining several aligned DOEs, made of different materials, similar to the polarization-selective DOEs [32, 33]. Furthermore, the flexibility of wavefront manipulation in different physical dimensions, e.g., phase, amplitude, wavelength, and polarization, in the metasurfaces makes it possible to encode multiple wavelength channels. For example, a designed metasurface consisting of different types of nanoblocks with spatially varying rotation angles multiplexed in a subwavelength unit can make it resonant with different wavelengths [34].

**Figure 5:**
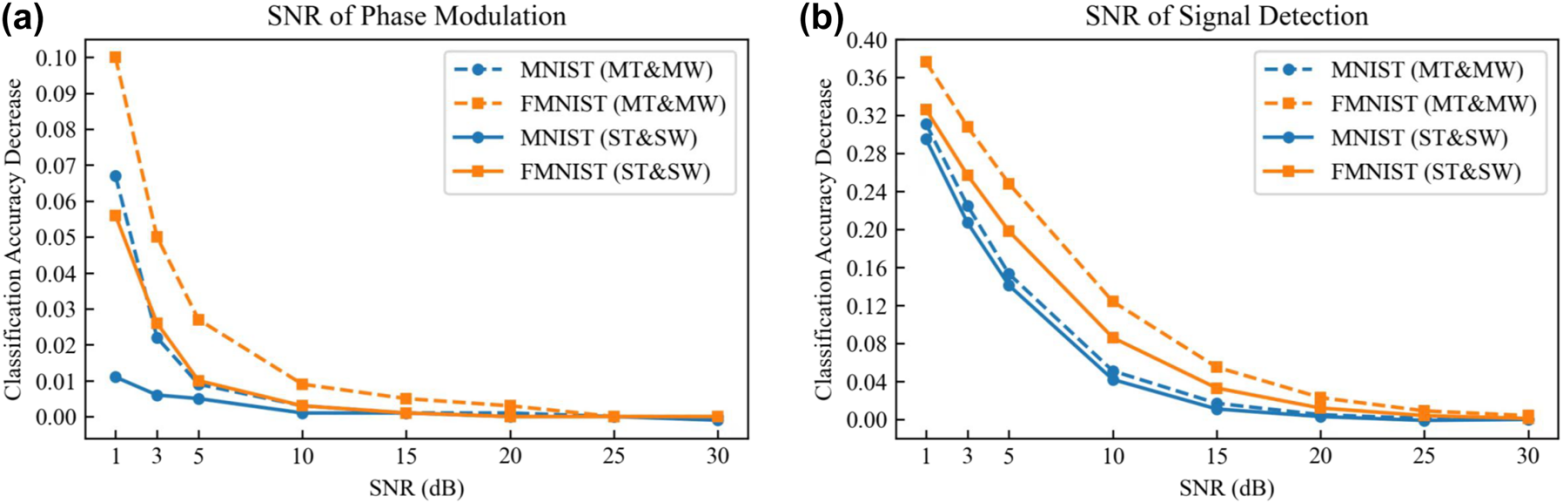
The comparisons of classification accuracy decrease between models of multiwavelength and multiple single-wavelength for multitask learning by incorporating different amounts of noise to the phase modulation (a) and the signal detection (b). MT and MW, multitask using multiwavelength; ST and SW, single-task using single-wavelength.

We analyze and compare the impact of phase modulation noise and signal detection noise, modeled with the Gaussian noise, on the classification accuracies of the multiwavelength D^2^NNs and single-wavelength D^2^NNs. We evaluate with the models of two-wavelength D^2^NN and two single-wavelength D^2^NNs to perform two tasks of classifying MNIST and fashion-MNIST databases (see Figure 3). The plots of classification accuracy decrease with respect to the SNR are shown in Figure 5a and b for the phase noise and detection noise, respectively. Numerical analysis results demonstrate that although training single-wavelength D^2^NN for each task has higher robustness at the low SNR settings, both multiwavelength and single-wavelength D^2^NNs achieve a slight classification accuracy decrease at the high SNR settings. When the SNR of the phase modulation is greater than 10 dB, or the SNR of the signal detection is greater than 25 dB, the accuracy loss of the two-wavelength D^2^NN and the single-wavelength D^2^NNs is less than 1%.

We further compare the training time of 1, 2, 3, and 4-wavelength D^2^NNs for 1, 2, 3, and 4-task learning, respectively, running on a single GPU, with the other network settings the same as the model in Figure 3. With the training epoch number setting of five, the time consumptions of four models are 116 s, 227 s, 365 s, and 425 s, respectively, which show approximate linear time growth with the increase of the wavelength numbers. Multiple GPUs for parallel computing can be easily utilized to reduce training time. Notice that the optical fields of different wavelengths propagate simultaneously in the multiwavelength physical system, which demands no additional time consumption during the inference of the forward model.

The multiwavelength D^2^NN is an all-optical computing processor that simultaneously performs multiple tasks with extremely high computational throughput. The computing speed of multiwavelength D^2^NN is limited by the encoding speed of input objects. Considering the experimental system architecture of a single-wavelength D^2^NN, the high-speed spatial light modulation (SLM) that works at ∼1000 fps in the visible spectrum typically can be used to encode the input images [8]. The SLM switching time is the bottleneck of the system, compared with the all-optical optical field propagation and detection rate; thus, the single-wavelength D^2^NN system can work at ∼1000 fps with the system latency of 1 ms in theory. For *N*-wavelength D^2^NN, the number of frames and operations processed per second can be increased by *N* times. Taking the four-wavelength D^2^NN configured with five layers and 800 × 800 modulation elements at each layer in Figure 4 as an example, similar to the calculations in [14], the total operation number of the optical forward model with single and four wavelengths are 324.4 million and 1.3 billion, respectively. As the DOEs are passive optical elements, with 10 mW of input light source power, the four-wavelength D^2^NN system has a computing energy efficiency of 130.0 Tera operations per second per watt (TOPS/W). Besides, compared with the spatial multiplexing of multiple D^2^NNs, the optical signals of different wavelength channels are independent of each other without any crosstalk during the multiwavelength diffractive optical computing. Therefore, increasing the wavelength channels in a single monolithic system increases the computing throughput and facilitates more tasks.

Nowadays, artificial neural networks still cannot learn in a continuous manner like mammalian brains, which is a great hindrance to the development of general artificial intelligence. It is widely accepted that catastrophic forgetting is a necessary flaw in the connectionist model. Although machine learning algorithms, such as transfer learning, focus on storing knowledge gained while solving one problem and applying it to different but related problems, its essence is a mathematical process that ignores physical properties and can only achieve the serial processing of different tasks. Multiwavelength D^2^NN has the inherent advantages of parallel processing of multiple tasks with light-speed processing, low power consumption, and high throughput. By encoding different tasks into different wavelength channels, multiwavelength D^2^NN can significantly alleviate competition among different tasks and maintain high performance for each task. For each new task, a new wavelength channel can be added easily to implement the new task. The task expansion process is shown in Figure 3A, and the cost of the whole process is extremely low. Therefore, multiwavelength D^2^NN can take full advantage of photonic computing and is expected to support realizing more general brain-inspired intelligence architecture in the future.

## 5 Conclusions

In this work, we have demonstrated the capability of multiwavelength D^2^NNs to achieve high-parallel classification and enable high-accuracy optical multitask learning with the joint optimization training method. By encoding multitasks into multiwavelength channels to exploit the wavelength dimension of the diffractive optical field, the proposed optical multitask learning approach can realize different tasks in parallel at the speed of light. The optical multitask function is implemented within a monolithic system and does not require the mechanical movement of diffractive modulation layers, significantly reducing the system’s complexity. Analysis reveals that the proposed method can significantly alleviate the competition between multitasks and maintain the performance of each task. As the task number increases, the multiwavelength D^2^NNs show greater advantages in realizing optical multitask learning. The proposed approach can be extended to other photonic neural network architectures by using the wavelength-division multiplexing technology to perform optical multitask learning that simultaneously achieves the capability of high-parallel, high-accuracy, and high-generality.
